# CD44 and Snail1 Expression Predicts Poor Prognosis of Oral Squamous Cell Carcinoma

**DOI:** 10.1111/jop.70032

**Published:** 2025-08-12

**Authors:** Cintia Eliza Marques, Everton Freitas de Morais, Bruno Cesar da Costa, Fábio Haach Téo, Ana Lúcia Carrinho Ayroza Rangel, Ricardo D. Coletta, Lívia Maris Ribeiro Paranaiba Dias

**Affiliations:** ^1^ Graduate Program in Oral Biology, School of Dentistry University of Campinas São Paulo Brazil; ^2^ Department of Oral Diagnosis, School of Dentistry University of Campinas São Paulo Brazil; ^3^ Center of Biological Sciences and of the Health, School of Dentistry State University of Western Paraná Cascavel Brazil; ^4^ Department of Pathology and Parasitology, Institute of Biomedical Sciences Federal University of Alfenas Alfenas Brazil

**Keywords:** cancer stem cells, epithelial‐mesenchymal transition, oral cancer, prognostic markers

## Abstract

**Background:**

Oral squamous cell carcinoma (OSCC) remains a challenging malignancy with poor 5‐year survival rates due to diagnosis at an advanced stage and a high likelihood of recurrence and metastasis. These aggressive traits may be influenced by cancer stem cells (CSC) and epithelial‐mesenchymal transition (EMT).

**Methods:**

This study investigated the prognostic significance of the CSC marker CD44 and EMT‐related proteins (Snail1, Snail2, E‐cadherin, N‐cadherin) in 132 OSCCs using immunohistochemistry. The comprehensive survival analysis included univariate and multivariate (stepwise method) Cox regression for disease‐specific survival (DSS) and disease‐free survival (DFS), Kaplan–Meier curves based on log‐rank testing, and receiver operating characteristic (ROC) analysis to assess the predictive accuracy of the markers.

**Results:**

High CD44 expression independently predicted worse DSS (HR = 2.74, 95% CI 1.44–5.23, *p* = 0.003) and DFS (HR = 2.22, 95% CI 1.16–4.23, *p* = 0.01), and Snail1 was significantly associated with poor DSS (HR = 2.62, 95% CI 1.37–5.03, *p* = 0.004). The combined expression of CD44 and Snail1 improved the discrimination of worse outcomes compared to markers individually. The presence of lymphovascular invasion (HR = 8.68, 95% CI 3.81–19.75, *p* < 0.0001) and a positive surgical margin (< 5 mm; HR = 4.45, 95% CI 1.99–9.96, *p* = 0.0003) were also independently associated with DSS.

**Conclusions:**

The results of this study highlight the prognostic significance of CD44 and Snail1 in OSCC, emphasizing their potential interplay in tumor aggressiveness.

## 1 Introduction

Oral squamous cell carcinoma (OSCC) is the most common malignant neoplasm of the oral cavity, predominantly affecting the lateral border of the oral tongue, primarily linked to cigarette smoking, and the buccal mucosa, where it is frequently associated with chewing tobacco [1]. Despite therapeutic advances, the 5‐year survival rate for patients with OSCC remains poor, with an average of approximately 50% [2]. Moreover, in its advanced stages, OSCC is associated with a highly unfavorable prognosis and 20%–40% of patients develop recurrence after treatment [3]. Consequently, the discovery of innovative biomarkers for early diagnosis, post‐treatment surveillance, and the advancement of new therapeutic strategies is critically important.

The low survival rate of OSCC patients may be attributed to the tumor's high genetic and biological complexity, which is primarily reflected in cellular heterogeneity, influencing development, progression, and therapeutic response [4]. Among the various cellular subpopulations in OSCC, cancer stem cells (CSCs) and cells undergoing epithelial‐mesenchymal transition (EMT) hold particular relevance. CSCs, characterized by their capacity for self‐renewal, differentiation, and high tumorigenic potential, play critical roles in tumor initiation, progression, metastasis, recurrence, and treatment resistance [5]. Likewise, EMT represents a fundamental process in tumor progression, enabling epithelial cells to acquire mesenchymal traits such as enhanced motility, invasiveness, and resistance to therapies, including immunotherapy [6]. Interactions with the tumor microenvironment can modulate CSCs, among them their ability to undergo EMT [7]. Although EMT is often viewed as a process that generates or sustains CSCs, contributing to tumor heterogeneity and increased metastatic potential, emerging studies point to a bidirectional relationship, in which CSCs can also induce and sustain the EMT state [8]. Indeed, growing evidence suggests that CSCs are functionally linked to EMT, expanding our understanding of tumor plasticity. Comprehending the intrinsic mechanisms connecting CSCs and EMT could lead to improved therapeutic strategies and prognostic outcomes in OSCC.

This study aimed to investigate the impact of the CSC marker CD44 and key EMT markers (N‐cadherin, E‐cadherin, Snail1, and Snail2, previously named as Slug) in the prognosis of patients with OSCC. Our findings indicate that CD44 and Snail1 overexpression is significantly associated with poor prognosis, with patients exhibiting high expression of both markers experiencing even shorter survival.

## 2 Materials and Methods

### 2.1 Cohort

The study examined a cohort of 132 histologically confirmed OSCC cases, which was previously described by us [9]. All patients underwent radical surgical resection as primary treatment, with some receiving adjuvant radiotherapy and/or chemotherapy. Surgical margins were measured as the shortest distance between the tumor and resection edge and classified using a 5 mm cutoff (> 5 mm vs. ≤ 5 mm). Histopathological features including tumor‐stroma ratio (TSR) and tumor budding (TB) were previously described by Dourado et al. [10], while the presence of perineural invasion (PNI) and lymphovascular invasion (LVI) was reported by Dolens et al. [9]. Post‐treatment monitoring included a minimum 5‐year follow‐up or until death. Recurrences were histopathologically confirmed. Primary endpoints were disease‐specific survival (DSS), representing the period from treatment initiation to disease‐related death or last follow‐up, and disease‐free survival (DFS), corresponding to the period from treatment initiation to recurrence (local, regional, or distant) or last recurrence‐free follow‐up. The study was approved by the institutional Research Ethics Committee (protocol CAAE: 55927322.0.0000.5418) and was conducted in accordance with national and international ethical guidelines for human research.

### 2.2 Immunohistochemistry

The sections (3 μm) were deparaffinized, rehydrated, and subjected to heat‐induced epitope retrieval in 10 mmol/L citrate buffer (pH 6.0) using pressurized decloaking. After endogenous peroxidase inactivation with 3% hydrogen peroxide, the sections were incubated with primary antibodies overnight at 4°C, followed by the EnVision Flex system (Agilent Technologies, Santa Clara, CA, USA). The primary antibodies were polyclonal rabbit anti‐CD44 (Sigma‐Aldrich, Saint Louis, MO, USA, clone P16070, diluted 1:1000), polyclonal rabbit anti‐Snail1 (Sigma‐Aldrich, Saint Louis, MO, USA, clone CL3700, diluted 1:50), polyclonal rabbit anti‐Snail2 (Abcam Cambridge, UK, clone O43623, diluted 1:400), polyclonal rabbit anti‐E‐Cadherin (Sigma‐Aldrich, Saint Louis, MO, USA, clone P12830, diluted 1:250), and monoclonal rabbit anti‐N‐Cadherin (Sigma‐Aldrich, Saint Louis, MO, USA, clone RM259, diluted 1:100). Reactions were developed with 3,3′‐diaminobenzidine tetrahydrochloride (Agilent Technologies, Santa Clara, CA, USA) and counterstained with hematoxylin. Positive and negative controls were performed.

### 2.3 Immunohistochemical Assessment

Immunoreactivity was quantified using a semi‐quantitative scoring system evaluating both the percentage of positive tumor cells and staining intensity [11]. Scores for the percentage of positive tumor cells were assigned as: 0 (negative, 0%), 1 (≤ 25% of positive cells), 2 (26%–50% of positive cells), 3 (51%–75% of positive cells), or 4 (> 75% of positive cells). Staining intensity was graded as: 0 (negative), 1 (weak), 2 (moderate), or 3 (strong). The final score was determined by summing the scores for the percentage of positive tumor cells and staining intensity, yielding from 0 to 7, which was further classified into low and high expression based on the median value.

The quantification was performed by two trained examiners, blinded to clinical outcomes. To assess intraobserver reproducibility, a randomly selected subset (*n* = 21) underwent duplicate evaluation with a 14‐day washout period. Inter‐rater reliability analysis using Cohen's Kappa coefficient (κ) demonstrated consistent agreement across all markers: Snail1 (κ = 0.97), Snail2 (κ = 0.95), E‐Cadherin (κ = 0.92), N‐cadherin (κ = 0.93), and CD44 (κ = 1.0).

### 2.4 Statistical Analysis

All statistical analyses were performed using the MedCalc Statistical Software (version 20.218). Both univariate and multivariate Cox regression models were used for survival analysis. Survival curves were estimated using the Kaplan–Meier method and compared using the log‐rank test. The prognostic performance was assessed via receiver operating characteristic (ROC) analysis, with discriminative capacity quantified by the area under the curve (AUC) including 95% confidence intervals. A *p*‐value ≤ 0.05 was considered statistically significant.

## 3 Results

### 3.1 Sample Characterization

The cohort comprised 132 OSCC patients with a mean age of 61.1 ± 13.3 years (range from 17 to 92 years). Demographic and clinical characteristics revealed male predominance (*n* = 94, 71.2%), frequent tobacco use (*n* = 87, 65.9%), alcohol consumption (*n* = 56, 42.4%), and advanced clinical stage at diagnosis (Stage IV: *n* = 43, 32.6%). The tongue was the most common primary site (*n* = 73, 55.3%), and tumor grading assessment identified moderately differentiated tumors in 69 cases (52.3%). Complete clinicopathological characteristics are detailed in Table S1.

### 3.2 Expression Patterns of the Markers

**FIGURE 1:**
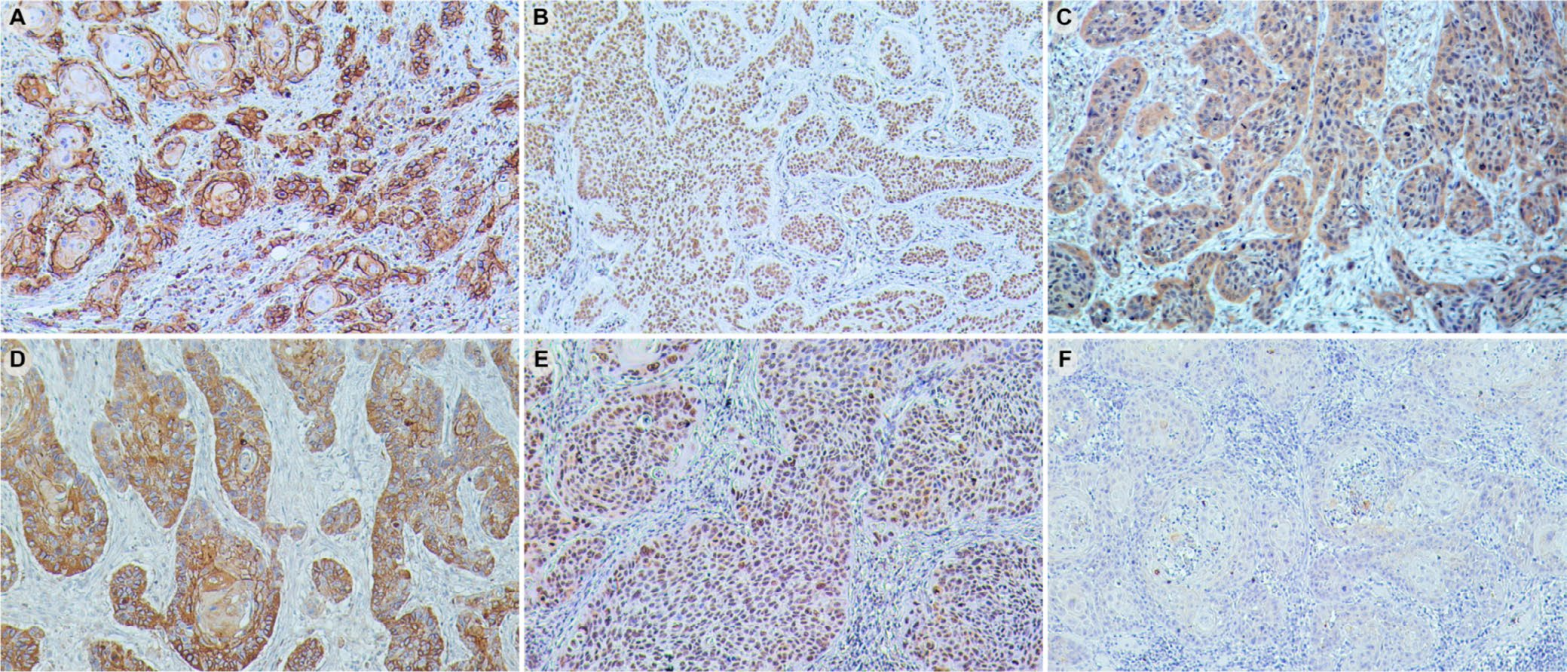
Immunohistochemical detection of CD44, Snail1, Snail2, E‐Cadherin, and N‐Cadherin in OSCC. Representative immunohistochemical images of each marker are depicted. (A) CD44 was mainly localized on the membrane of the tumor cells. (B) Snail1 expression was concentrated in the nuclei of the tumor cells. (C) The expression of Snail2 was detected predominantly in the cytoplasm, with some tumor cells also exhibiting nuclear expression. (D) E‐Cadherin immunoexpression was observed in both the cytoplasm and membrane of the tumor cells. (E) N‐Cadherin showed variable staining intensity in both the nucleus and cytoplasm of tumor cells. (F) Negative control, in which the reaction was performed by omission of the primary antibody. Original magnification ×100.

CD44 was identified mainly in the membrane and cytoplasm of the tumor cells, but with variable intensity (Figure 1A). Positivity for Snail1 was identified exclusively as nuclear stain with variable distribution and intensity in the tumor cells (Figure 1B). Snail2 expression was observed with variable intensity in the cytoplasm of tumor cells, with some cells displaying nuclear stain (Figure 1C). E‐Cadherin showed a heterogeneous expression pattern, with variable intensity in both membranous and cytoplasmic locations (Figure 1D). Regarding N‐Cadherin, variable staining intensity was observed in both the nucleus and cytoplasm of tumor cells (Figure 1E). A representative image was used as a negative control, showing no immunolabeling in the tissue section (Figure 1F).

### 3.3 CD44 and Snail1 Are Independently Associated With DSS

Univariate survival analysis for DSS showed a significant association between poor prognosis and several factors, including alcohol consumption (HR = 2.13, 95% CI 1.14–4.01, *p* = 0.02), clinical stage (HR = 2.15, 95% CI 1.14–4.06, *p* = 0.02), surgical margins (HR = 2.16, 95% CI 1.07–4.35, *p* = 0.03), PNI (HR = 2.35, 95% CI 1.36–4.06, *p* = 0.002), LVI (HR = 2.77, 95% CI 1.44–5.34, *p* = 0.002), TSR (HR = 2.58, 95% CI 1.41–4.70, *p* = 0.002), and CD44 (HR = 2.35, 95% CI 1.32–4.18, *p* = 0.004), and Snail1 expression (HR = 2.00, 95% CI 1.13–3.53, *p* = 0.017) (Table 1). Kaplan–Meier curves showed that tumors with high expression of CD44 (Figure S1) or Snail1 (Figure S1B) displayed statistically poorer DSS compared with low‐expressing tumors.

**TABLE 1:** Univariate and multivariate analysis for disease‐specific survival (DSS) of patients with oral squamous cell carcinoma.

	Univariate analysis		Multivariate analysis	
	HR (95% CI)	*p*	HR (95% CI)	*p*
Age (years)				
≥ 62	1			
< 62	1.28 (0.74–2.22)	0.37		
Sex				
Male	1			
Female	0.60 (0.31–1.16)	0.13		
Alcohol consumption				
No	1			
Yes	2.13 (1.14–4.01)	**0.02**		
Smoking				
No	1			
Yes	0.94 (0.47–1.86)	0.86		
Clinical stage				
Initial (I + II)	1			
Advanced (III + IV)	2.15 (1.14–4.06)	**0.02**		
Tumor site				
Tongue	1			
Floor of mouth	1.16 (0.55–2.42)	0.70		
Other sites	1.32 (0.97–1.81)	0.07		
Treatment				
Surgery	1			
Surgery + radiotherapy	1.54 (0.83–2.90)	0.17		
Surgery + radiotherapy + chemotherapy	1.00 (0.70–1.43)	0.99		
Margin status				
> 5 mm	1		1	
≤ 5 mm	2.16 (1.07–4.35)	**0.03**	4.45 (1.99–9.96)	**0.0003**
WHO histopathological grading				
Well differentiated	1			
Moderately differentiated	1.06 (0.58–1.92)	0.84		
Poorly differentiated	1.27 (0.82–1.97)	0.30		
Tumor budding (TB)				
No (< 5 buds)	1			
Yes (≥ 5 buds)	1.60 (0.91–2.74)	0.10		
Perineural invasion (PNI)				
No	1			
Yes	2.35 (1.36–4.06)	**0.002**		
Lymphovascular invasion (LVI)				
No	1		1	
Yes	2.77 (1.44–5.34)	**0.002**	8.68 (3.81–19.75)	**< 0.0001**
Tumor–stroma ratio (TSR)				
< 50%	1			
≥ 50%	2.58 (1.41–4.70)	**0.002**		
CD44				
Low expression	1		1	
High expression	2.35 (1.32–4.18)	**0.004**	2.74 (1.44–5.23)	**0.002**
Snail1				
Low expression	1		1	
High expression	2.0 (1.13–3.53)	**0.017**	2.62 (1.37–5.03)	**0.004**
Snail2 (Slug)				
Low expression	1			
High expression	0.83 (0.46–1.50)	0.54		
E‐Cadherin				
Low expression	1			
High expression	1.19 (0.68–2.07)	0.54		
N‐Cadherin				
Low expression	1			
High expression	1.0 (0.57–1.72)	0.99		

To determine whether these factors serve as independent markers of DSS, a multivariate survival analysis based on the stepwise model of the Cox regression test was employed. The results identified surgical margins (HR = 4.45, 95% CI 1.99–9.96, *p* = 0.0003), LVI (HR = 8.68, 95% CI 3.81–19.75, *p* < 0.0001), CD44 expression (HR = 2.74, 95% CI 1.44–5.23, *p* = 0.002), and Snail1 expression (HR = 2.62, 95% CI 1.37 to 5.03, *p* = 0.004) as independent prognostic factors for DSS (Table 1).

### 3.4 CD44 Expression is Associated With DFS

In the univariate analysis, male gender (HR = 0.49, 95% CI 0.25–0.65, *p* = 0.03), high TSR (HR = 1.81, 95% CI 1.03–3.15, *p* = 0.04), and high CD44 expression (HR = 2.12, 95% CI 1.15–3.91, *p* = 0.01) were predictive of poorer DFS (Table 2). However, at the multivariate Cox analysis, only high CD44 expression remained an independent prognostic factor (HR = 2.22, 95% CI 1.16–4.23, *p* = 0.01). Kaplan–Meier curve further confirmed that patients with high CD44 expression exhibited a significantly increased risk of recurrence (HR = 2.43, 95% CI 1.21–4.86, *p* = 0.01) (Figure S1C).

**TABLE 2:** Univariate and multivariate analysis for disease‐free survival (DFS) of patients with oral squamous cell carcinoma.

	Univariate analysis		Multivariate analysis	
	HR (95% CI)	*p*	HR (95% CI)	*p*
Age (years)				
≥ 62	1			
< 62	1.18 (0.68–2.03)	0.54		
Sex				
Male	1			
Female	0.49 (0.25–0.65)	**0.03**		
Alcohol consumption				
No	1			
Yes	1.04 (0.58–1.86)	0.88		
Smoking				
No	1			
Yes	1.13 (0.56–2.28)	0.76		
Clinical stage				
Initial (I + II)	1			
Advanced (III + IV)	1.64 (0.91–2.95)	0.09		
Tumor site				
Tongue	1			
Floor of mouth	1.04 (0.49–2.20)	0.91		
Other sites	1.15 (0.84–1.51)	0.37		
Treatment				
Surgery	1			
Surgery + radiotherapy	1.16 (0.59–2.24)	0.66		
Surgery + radiotherapy + chemotherapy	1.18 (0.85–1.65)	0.31		
Margin status				
> 5 mm	1			
≤ 5 mm	1.50 (0.72–3.08)	0.27		
WHO histopathological grading				
Well differentiated	1			
Moderately differentiated	1.04 (0.49–2.20)	0.91		
Poorly differentiated	1.15 (0.84–1.51)	0.37		
Tumor budding (TB)				
No (< 5 buds)	1			
Yes (≥ 5 buds)	1.32 (0.75–2.31)	0.32		
Perineural invasion (PNI)				
No	1			
Yes	1.61 (0.93–2.78)	0.08		
Lymphovascular invasion (LVI)				
No	1			
Yes	1.22 (0.55–2.71)	0.62		
Tumor–stroma ratio (TSR)				
< 50%	1			
≥ 50%	1.81 (1.03–3.15)	**0.04**		
CD44				
Low expression	1		1	
High expression	2.12 (1.15–3.91)	**0.01**	2.22 (1.16–4.23)	**0.01**
Snail1				
Low expression	1			
High expression	1.46 (0.82–2.60)	0.20		
Snail2 (Slug)				
Low expression	1			
High expression	0.98 (0.52–1.84)	0.96		
E‐Cadherin				
Low expression	1			
High expression	0.77 (0.4–1.43)	0.42		
N‐Cadherin				
Low expression	1			
High expression	0.79 (0.45–1.37)	0.40		

### 3.5 Combined Expression of CD44 and Snail1 is Associated With Both DSS and DFS

**FIGURE 2:**
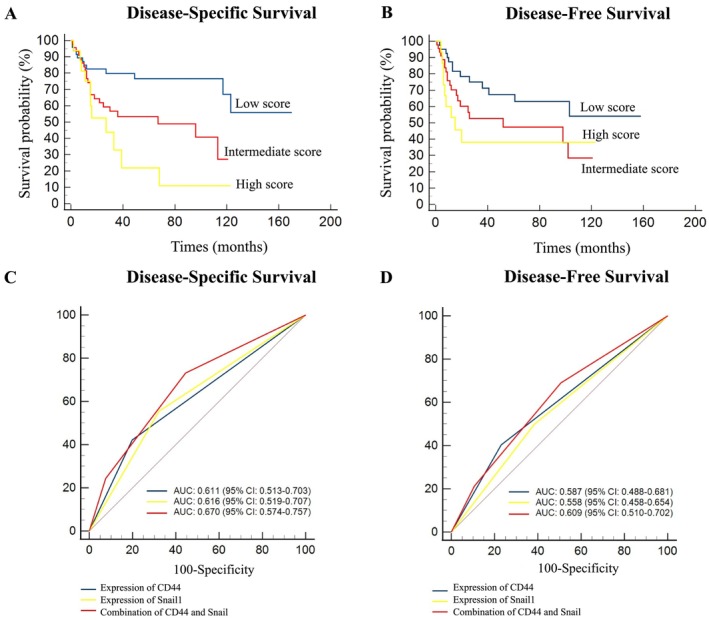
Survival curves and receiver operating characteristic (ROC) curves based on combined CD44 and Snail1 expression score. (A) Kaplan–Meier survival disease‐specific survival and (B) disease‐free survival. (C) ROC curve evaluating disease‐specific survival and (D) ROC curve evaluating disease‐free survival.

In order to explore the prognostic value of immunohistochemical CD44 and Snail1 expression, which were independently associated with patient outcomes, these markers were combined and subjected to univariate and multivariate survival analysis. Based on their expression levels, tumors were categorized as follows: low risk—tumors with both low expression of CD44 and Snail1, intermediate risk—tumors with low expression of CD44 and high expression of Snail1 or tumors with high expression of CD44 but low expression of Snail1, and high risk—tumors with high expressions of CD44 and Snail1. Significant associations were observed between the combinations of CD44 and Snail1 and both DSS and DFS (Table 3). Compared to patients classified as low risk, patients classified as intermediate risk (HR = 2.39, 95% CI 1.49–3.81, *p* = 0.002) and high risk (HR = 3.57, 95% CI: 1.57–8.11, *p* = 0.0003) had significantly shortened DSS. A similar trend was observed for DFS, where patients classified as intermediate risk (HR = 1.68, 95% CI 1.08–2.60, *p* = 0.02) and high risk (HR = 2.20, 95% CI 1.11–4.50, *p* = 0.01) displayed a higher risk of recurrence than patients classified as low risk (Table 3). In all combinations, the Kaplan–Meier survival curves demonstrated an improved discriminatory ability (Figure 2A,B).

**TABLE 3:** Cox regression analysis for disease‐specific survival and disease‐free survival for combination of CD44 and Snail1 expressions in the 132 oral squamous cell carcinoma patients.

Parameters	Disease specific survival		Disease‐free survival	
	Hazard ratio (95% CI)/*p*‐value		Hazard ratio (95% CI)/*p*‐value	
	Univariate	Multivariate	Univariate	Multivariate
CD44 and Snail1				
Low risk	1	1	1	1
Intermediate risk	1.93 (1.27–2.93)/0.01	2.39 (1.49–3.81)/0.002	1.70 (0.99–2.63)/0.06	1.68 (1.08–2.60)/0.02
High risk	2.48 (1.19–5.14)/0.002	3.57 (1.57–8.11)/0.0003	2.01 (1.10–4.06)/0.02	2.20 (1.11–4.50)/0.01

*Note*: Low risk represents the tumors with low expression of both markers, Intermediate risk represents either low for CD44 and high for Snail1 or high for CD44 and low for Snail1, and High risk represents the tumors with high expression of both markers.

To further evaluate the predictive power of the model, the AUCs of ROC curves were compared. As depicted in Figure 2C, the model provided superior discrimination for DSS (AUC = 0.670, 95% CI 0.574–0.757) compared to individual marker expressions. Although less pronounced, the model also showed higher discrimination (AUC = 0.609, 95% CI 0.510–0.702) for DFS than CD44 or Snail1 individually (Figure 2D).

## 4 Discussion

The clinical management of OSCC remains challenging due to its heterogeneous biological behavior. The clinical staging (TNM system) serves as the gold standard for prognostic evaluation and treatment planning, but even early‐stage tumors may demonstrate unexpectedly aggressive progression [12]. Similarly, assessing classical histological features such as PNI, LVI, and margin status, or new ones including TB and TSR has proven valuable for risk stratification, determining optimal therapeutic protocol, and survival prediction of OSCC. However, the inconsistencies in the definitions and scales/cut‐off have limited their daily use [13]. These aspects underscore the critical need for reliable biomarkers that could improve early detection accuracy and therapeutic monitoring [4]. The findings of this study demonstrated that high expression of CD44 and Snail1 was significantly associated with reduced survival. Snail1 emerged as an independent prognostic factor for DSS, whereas CD44 showed a relevant impact on both DSS and DFS, which is consistent with findings from other studies [14, 15, 16]. The results also revealed that the combination of CD44 and Snail1 provided superior discrimination of the endpoints than the markers alone, suggesting a possible functional interaction between these markers in OSCC. Indeed, this association is consistent with findings of studies with other cancer types. In ovarian cancer, the co‐expression of CD44 and Snail1 was linked to tumor progression and poorer prognosis [14]. Similarly, overexpression of CD44 induced Snail1 expression, but not Twist1 or Zeb1, promoting phenotypes consistent with EMT in pancreatic cancers [17]. Furthermore, CD44 inhibition led to reduced Snail1 levels, while suppression of the epithelial splicing regulatory protein 1 (ESRP1), a regulator of CD44 splicing, increased the expression of both CD44 and Snail1 [18]. These findings are also supported by studies in breast cancer, where ESRP1‐mediated CD44 isoform switching was essential for EMT induction and tumor progression [18]. In an in vitro study, CD44 was found to promote tumor progression through EMT by regulating Snail1 expression, supporting a functional relationship in which CD44 acts as an upstream regulator of EMT [14]. Collectively, these findings suggest that the CD44 –Snail1 axis plays an important role in connecting CSC and EMT, influencing therapeutic response and prognosis across various tumors.

The nuclear transcription factor Snail1 is a master regulator of EMT across multiple cancer types [19]. In ovarian carcinomas, high Snail1 expression was associated with high‐grade tumors and the presence of metastasis, impacting the response to therapy and survival rates of the patients [20]. Similar results have been reported in other cancer types, including breast, lung, and gastric tumors [21, 22, 23]. Snail1 involvement in OSCC pathogenesis is under constant investigation, and the findings of the present study point to its prognostic relevance. Our previous study demonstrated that the elevated Snail1 expression throughout oral tongue carcinogenesis stages is inversely correlating with membranous E‐cadherin expression, and Snail1 silencing effectively inhibited the migration and invasion and suppressed EMT of OSCC cells [11]. Moreover, Snail1 contributed to CSC‐like properties in OSCC by driving EMT, thereby enhancing tumor sphere formation, chemoresistance, and invasive potential [24].

Beyond EMT, research on cellular plasticity has increasingly focused on identifying CSC‐associated prognostic markers. Although CSCs represent a minor tumor subpopulation, they possess unique tumor‐initiating capacity and drive cancer recurrence and therapy resistance [25]. CSCs possess the ability to generate populations with dynamic cellular states that can transition between epithelial and mesenchymal phenotypes. These transitions are influenced by tumor microenvironmental factors, including inflammatory signaling, hypoxia, and interactions with stromal cells [26]. The regulation of CSC plasticity involves intricate signaling pathways and multiple non‐coding RNAs, which directly affect self‐renewal capacity and contribute to intratumoral heterogeneity [27]. Our findings establish CD44 as a clinically relevant prognostic marker for survival and recurrence risk in OSCC. A recent meta‐analysis on CD44 expression in OSCC found that high CD44 levels are significantly associated with clinicopathological features linked to tumor aggressiveness, including regional and distant metastases, advanced clinical stage, and poor tumor differentiation, leading to shortened survival and an increased risk of recurrence [28]. However, CSC analysis remains methodologically challenging, requiring comprehensive marker panels to capture their phenotypic diversity within the tumor niche [29]. The inherent heterogeneity of CSCs and their dynamic crosstalk with the tumor microenvironment critically influence disease progression and therapeutic outcomes. Moving forward, research efforts should prioritize standardizing robust CSC detection protocols and validating the clinical utility of CD44 and other CSC markers to optimize personalized OSCC management strategies.

Pathological parameters such as surgical margins and LVI have proven to be reliable indicators of DSS. Achieving clear margins is the primary goal in the surgical management of OSCC as it is associated with a reduced risk of recurrence and improved survival. Conversely, a positive margin or a margin with an inadequate distance of normal tissue has negative prognostic implications, necessitating adjuvant treatment [30]. Currently, a clear resection margin is commonly defined as greater than 5 mm [31]. Several systematic reviews and meta‐analyses have examined tumor margin status and prognosis, with the largest study to date demonstrating a strong association between the positive tumor margin and shortened overall survival, DSS, and DFS [13]. LVI plays a critical role in tumor metastasis and has been widely recognized as a prognostic marker for poor outcomes in OSCC [32, 33]. A comprehensive systematic review and meta‐analysis, encompassing 30 studies and over 30 000 samples, highlighted the significant prognostic role of LVI in OSCC [13]. However, some studies have challenged this assertion, suggesting that LVI may not have meaningful prognostic value in OSCC [34, 35]. Possible explanations for these conflicting findings include the heterogeneity of OSCC biology, challenges in identifying LVI in hematoxylin and eosin‐stained slides, and inconsistencies in the definition of LVI. Some studies define LVI strictly as the presence of tumor cells within the vascular space, whereas others classify it more broadly, including tumor cells both within or adjacent to the vessels.

Together, our findings indicate that CD44 and Snail1 represent markers for OSCC prognosis. In contrast, no significant association was found for other EMT markers examined in this study (Snail2, E‐Cadherin and N‐Cadherin). The interpretations of the current findings should be taken with caution, and future investigations should integrate multimodal analytical strategies to elucidate marker interactions and therapeutic potential. Additionally, prospective investigations with larger cohorts, preferentially involving multiple centers and expanded biomarker panels, would deepen our understanding of these molecular mechanisms and their clinical applications in OSCC management, paving the way for targeted therapies.

## Author Contributions

**Cintia Eliza Marques:** conceptualization; data curation; formal analysis; investigation; methodology; writing‐original draft; writing – review and editing. **Everton Freitas de Morais:** conceptualization; data curation; formal analysis; investigation; methodology; writing – original draft; writing – review and editing. **Bruno Cesar da Costa:** data curation; formal analysis; investigation; methodology; writing – review and editing. **Fábio Haach Téo:** data curation; formal analysis; writing – review and editing. **Ana Lúcia Carrinho Ayroza Rangel:** data curation; formal analysis; writing – review and editing. **Ricardo D. Coletta:** conceptualization; data curation; formal analysis; funding acquisition; investigation; methodology; project administration; supervision; writing – original draft; writing – review and editing. **Lívia Maris Ribeiro Paranaiba Dias:** conceptualization; data curation; formal analysis; funding acquisition; investigation; writing – review and editing.

## Ethics Statement

This study was conducted in accordance with the Declaration of Helsinki. The study protocol was approved by the National Research Ethics Committee (CAAE: 55927322.0.0000.5418).

## Conflicts of Interest

The authors declare no conflicts of interest.

## Peer Review

The peer review history for this article is available at https://www.webofscience.com/api/gateway/wos/peer‐review/10.1111/jop.70032.

## Supporting information


**Figure S1:** Kaplan–Meier survival curves for patients according to immunohistochemical parameters (**A**) Disease‐specific survival based on CD44 expression, (**B**) disease‐specific survival based on Snail1 expression, and (**C**) disease‐free survival based on CD44 expression

**Table S1:** Clinicopathological features of 132 patients with oral squamous cell carcinoma included in this study.

## Data Availability

The data to support the findings of this study will be available on request from the corresponding authors.
