# Supplementary material for: CD44 and Snail1 Expression Predicts Poor Prognosis of Oral Squamous Cell Carcinoma
Source: J Oral Pathol Med. 2025 Aug 12;54(9):835–45. doi: 10.1111/jop.70032 (PMC12521071; doi:10.1111/jop.70032)
Supplement: Supplementary file 1 — Figure S1: Kaplan–Meier survival curves for patients according to immunohistochemical parameters (A) Disease‐specific survival based on CD44 expression, (B) disease‐specific survival based on Snail1 expression, and (C) disease‐free survival based on CD44 expression [file JOP-54-835-s001.pdf]

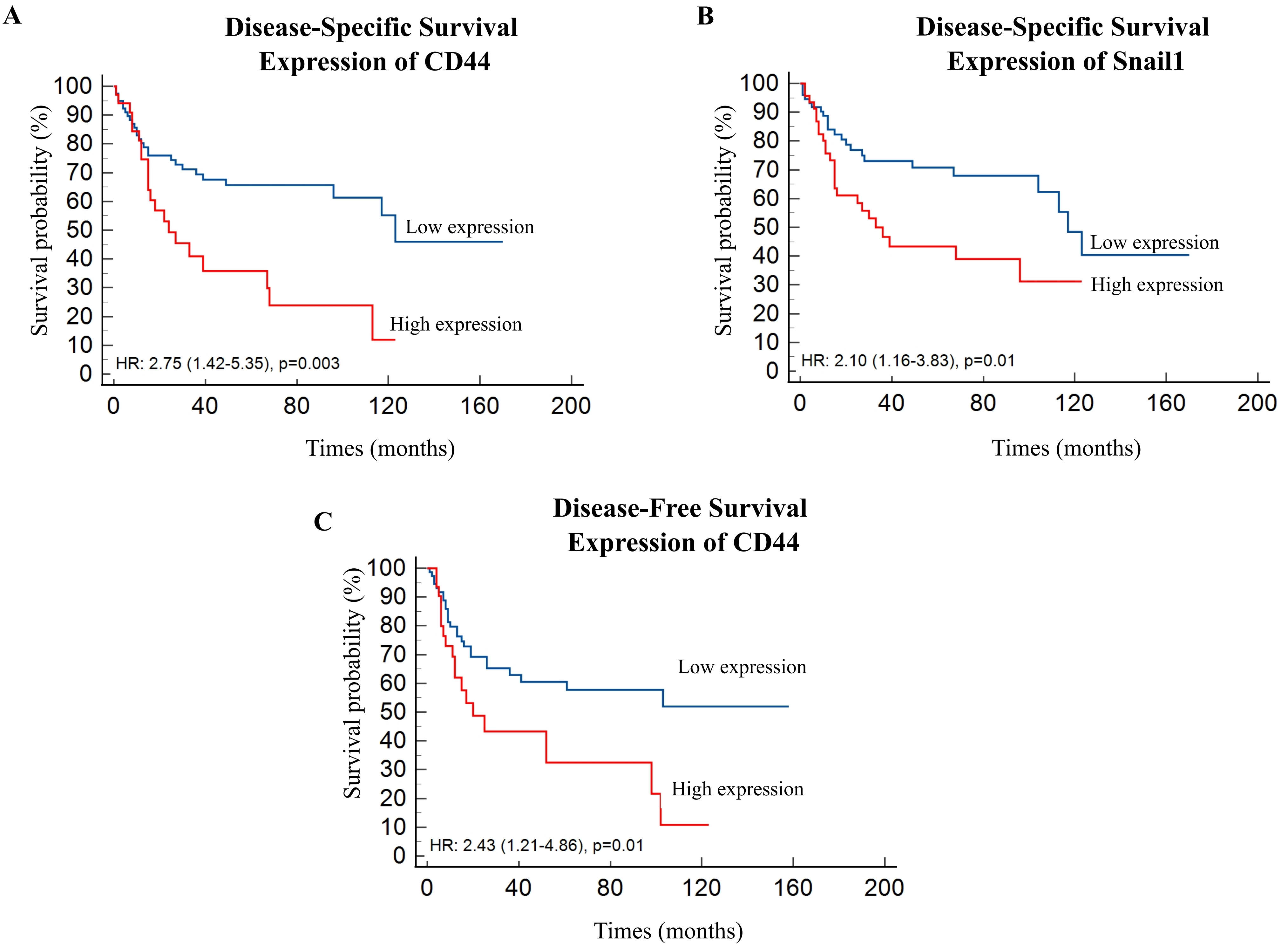
A
Disease-Specific Survival
Expression of CD44
Survival probability (%)
100
90
80
70
60
50
40
30
20
10
0
Low expression
High expression
HR: 2.75 (1.42-5.35), p=0.003
0
40
80
120
160
200
Times (months)
B
Disease-Specific Survival
Expression of Snail1
Survival probability (%)
Low expression
High expression
HR: 2.10 (1.16-3.83), p=0.01
Times (months)
C
Disease-Free Survival
Expression of CD44
Survival probability (%)
Low expression
High expression
HR: 2.43 (1.21-4.86), p=0.01
Times (months)
