# Supplementary material for: CD44 and Snail1 Expression Predicts Poor Prognosis of Oral Squamous Cell Carcinoma
Source: J Oral Pathol Med. 2025 Aug 12;54(9):835–45. doi: 10.1111/jop.70032 (PMC12521071; doi:10.1111/jop.70032)
Supplement: Supplementary file 2 — Table S1: Clinicopathological features of 132 patients with oral squamous cell carcinoma included in this study. [file JOP-54-835-s002.docx]

**Supplementary Table 1 │** Clinicopathological features of 132 patients with oral squamous cell carcinoma included in this study.

| Sample | N (%) |
| --- | --- |
| Total | 132 (100%) |
| Age (years) |  |
| Mean ± standard deviation | 61.1 ± 13.3 |
| Range | 17 - 92 |
| Sex |  |
| Male | 94 (71.2%) |
| Female | 38 (28.8%) |
| Alcohol consumption |  |
| No | 59 (44.7%) |
| Yes | 56 (42.4%) |
| Missing | 17 (12.9%) |
| Smoking |  |
| No | 27 (20.5%) |
| Yes | 87 (65.9%) |
| Missing | 18 (13.6%) |
| Clinical stage |  |
| I | 10 (7.6%) |
| II | 39 (29.5%) |
| III | 39 (29.5%) |
| IV | 43 (32.6%) |
| Missing | 1 (0.8%) |
| Tumor site |  |
| Tongue | 73 (55.3%) |
| Floor of mouth | 23 (17.4%) |
| Other sites |  |
| Retromolar trigone | 13 (9,9%) |
| Palate | 10 (7.6%) |
| Buccal mucosa | 7 (5.3%) |
| Gingiva | 6 (4.5%) |
| Treatment |  |
| Surgery | 53 (40.2%) |
| Surgery + Radiotherapy | 47 (35.6%) |
| Surgery + Radiotherapy + Chemotherapy | 32 (24.2%) |
| Margin status |  |
| >5 mm | 113 (85.6%) |
| ≤5 mm | 19 (14.4%) |
| WHO histopathological grading |  |
| Well differentiated | 49 (37.1%) |
| Moderately differentiated | 69 (52.3%) |
| Poorly differentiated | 14 (10.6%) |
| Tumor budding (TB) |  |
| No (<5 buds) | 85 (64.4%) |
| Yes (≥5 buds) | 35 (32.6%) |
| Perineural invasion (PNI) |  |
| No | 84 (63.6%) |
| Yes | 48 (36.4%) |
| Lymphovascular invasion (LVI) |  |
| No | 112 (84.8%) |
| Yes | 20 (15.2%) |
| Tumor–stroma ratio (TSR) |  |
| <50% | 65 (49.2%) |
| ≥50% | 67 (50.8%) |
| Local recurrence |  |
| No | 89 (67.4%) |
| Yes | 39 (28.8%) |
| Missing | 5 (3.8%) |
| Regional recurrence |  |
| No | 105 (79.6%) |
| Yes | 23 (17.4%) |
| Missing | 4 (3.0%) |
| Distant recurrence |  |
| No | 120 (91.0%) |
| Yes | 6 (4.5%) |
| Missing | 6 (4.5%) |
| Status |  |
| Alive | 80 (60.6%) |
| Dead | 52 (39.4%) |
